# Successful extracorporeal membrane oxygenation resuscitation of patient with cardiogenic shock induced by phaeochromocytoma crisis mimicking hyperthyroidism: A case report

**DOI:** 10.1515/biol-2021-0073

**Published:** 2021-07-16

**Authors:** Tao Wang, Qiancheng Xu, Xiaogan Jiang

**Affiliations:** Department of Critical Care Medicine, The First Affiliated Hospital of Wannan Medical College, Yijishan Hospital, Wuhu, China

**Keywords:** hyperthyroidism, phaeochromocytoma crisis, ECMO, cardiocirculatory system collapse, intensive care unit

## Abstract

A 29-year-old woman presented to the emergency department with the acute onset of palpitations, shortness of breath, and haemoptysis. She reported having an abortion (56 days of pregnancy) 1 week before admission because of hyperthyroidism diagnosis during pregnancy. The first diagnoses considered were cardiomyopathy associated with hyperthyroidism, acute left ventricular failure, and hyperthyroidism crisis. The young woman’s cardiocirculatory system collapsed within several hours. Hence, venoarterial extracorporeal membrane oxygenation (VA ECMO) was performed for this patient. Over the next 3 days after ECMO was established, repeat transthoracic echocardiography showed gradual improvements in biventricular function, and later the patient recovered almost completely. The patient’s blood pressure increased to 230/130 mm Hg when the ECMO catheter was removed, and then the diagnosis of phaeochromocytoma was suspected. Computed tomography showed a left suprarenal tumour. The tumour size was 5.8 cm × 5.7 cm with central necrosis. The vanillylmandelic acid concentration was 63.15 mg/24 h. Post-operation, pathology confirmed phaeochromocytoma. To our knowledge, this is the first case report of a patient with cardiogenic shock induced by phaeochromocytoma crisis mimicking hyperthyroidism which was successfully resuscitated by VA ECMO.

## 1 Introduction

Phaeochromocytoma arises from neuroectodermal tissue that secretes catecholamines and often presents various benign symptoms, such as headache, palpitations, and sweating, but can also present multiple organ failure. All kinds of acute diseases, such as acute coronary syndrome, cardiogenic shock, septic shock [1], preeclampsia, and amniotic fluid embolism [2], have been reported to be mimicked by phaeochromocytoma crisis (PCC). We are the first to report a patient with cardiogenic shock induced by PCC mimicking hyperthyroidism which was successfully resuscitated by venoarterial extracorporeal membrane oxygenation (VA ECMO).

## 2 Case report

A 29-year-old woman presented to the emergency department with the acute onset of palpitations, shortness of breath, and haemoptysis. She reported having an abortion (56 days of pregnancy) 1 week prior to admission because of a hyperthyroidism diagnosis during pregnancy; the thyroid function increased significantly (FT3: 7.03 pmol/L, FT4: 26.31 pmol/L, and TSH: 0.008 mIU/L). The patient denied using any drugs or tobacco, and there was no history of allergies. Her family history was normal. She was admitted to the endocrinology department under the consideration of cardiomyopathy associated with hyperthyroidism, acute left ventricular failure, and hyperthyroidism crisis as potential diagnoses.

**Figure 1:**
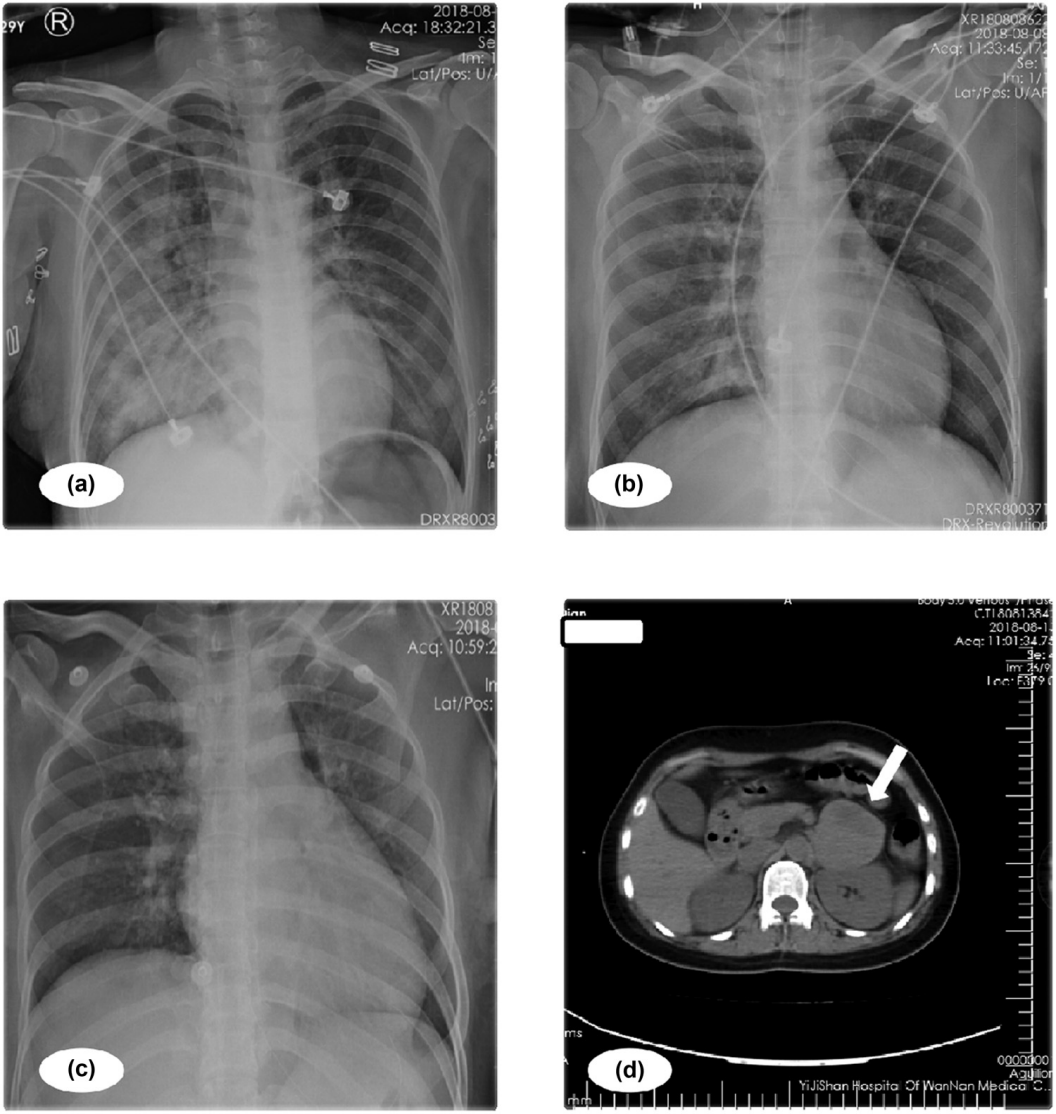
Radiologic examination of the patient. (a) Chest X-ray showing serious interstitial and alveolar oedema. (b and c) Chest posteroanterior shows interstitial and alveolar oedema improved. (d) CT revealed a left suprarenal tumour of 5.8 cm × 5.7 cm in size with necrosis (white arrow).

On admission, she was conscious but displayed acrocyanosis, orthopnoea, excessive perspiration, moist cold skin, cough with pink bubbly expectorate, and respiratory distress. Her respiratory rate was 40 breaths/min, her oxygen saturation (SPO_2_) by pulse oximetry was 93% with an oxygen delivery rate of 15 L/min by hydrogen mass, and her heart rate was 156 beats/min with hypotension of 85/43 mm Hg with a dopamine dose of 8 µg/kg min. This dose was increased slowly. Her shell temperature was 36.3°C. Abdominal examination revealed a soft abdomen with no organ enlargement or mass. She had auscultation of the lungs with wheezes or crackles. Laboratory studies showed hyperglycaemia (glucose: 9.43 mmol/L), myocardial damage (troponin I: 29.02 ng/mL and creatine kinase-MB: 64 U/mL), and mild renal insufficiency (creatinine: 146.4 mmol/L and anuria lasting for 3 h); her PCT level was 24 ng/mL and her CRP level was 2.0 mg/L. Transthoracic echocardiography (TTE) showed the normal left ventricular size and significantly reduced left ventricular systolic function. The left ventricular ejection fraction (LVEF) was 15%. Chest X-ray revealed severe interstitial and alveolar oedema (Figure 1a). The patient was administered propylthiouracil, esmolol, dopamine, furosemide, and morphine. The patient’s condition was then exacerbated; non-invasive mechanical ventilation was used, and she was transferred to the ICU. She was started on continuous renal replacement therapy with epinephrine and norepinephrine infusion to support myotonic and vasopressin. Over the next few hours, despite efforts to maintain tissue perfusion, the patient’s condition worsened. Her vital signs (HR: 158 bpm, SPO_2_: 96%, and BP: 94/54 mm Hg) remained unstable despite high dose of norepinephrine (2.0 µg/kg/min), adrenaline (1.0 µg/kg/min), and dopamine (20 µg/kg/min) with ventilator support (FIO2: 100% and PEEP: 15 cm H_2_O). Blood gas analysis showed severe hyperlacticaemia and metabolic acidosis (pH: 7.300, Lac: 3.2 mmol/L, and BE: −10.0 mmol/L). Echocardiography revealed that the EF and global ventricular wall motion were much worse than before. PICCO showed severely low cardiac output and hyperpyrexia (T: 40.1°C, CI: 1.67, SI: 10.9, SVRI: 2,437, and EVLWI: 7.8). She was considered to be having hyperthyroidism crisis-induced cardiogenic shock. Mechanical ventilation was started, and the haemofiltration solution temperature was decreased to room temperature. Two hours later, the patient was still hyperpyrexic (40.3°C) and abruptly developed severe bradycardia; intermittent intravenous epinephrine was administered to maintain her heart rate.

**Figure 2:**
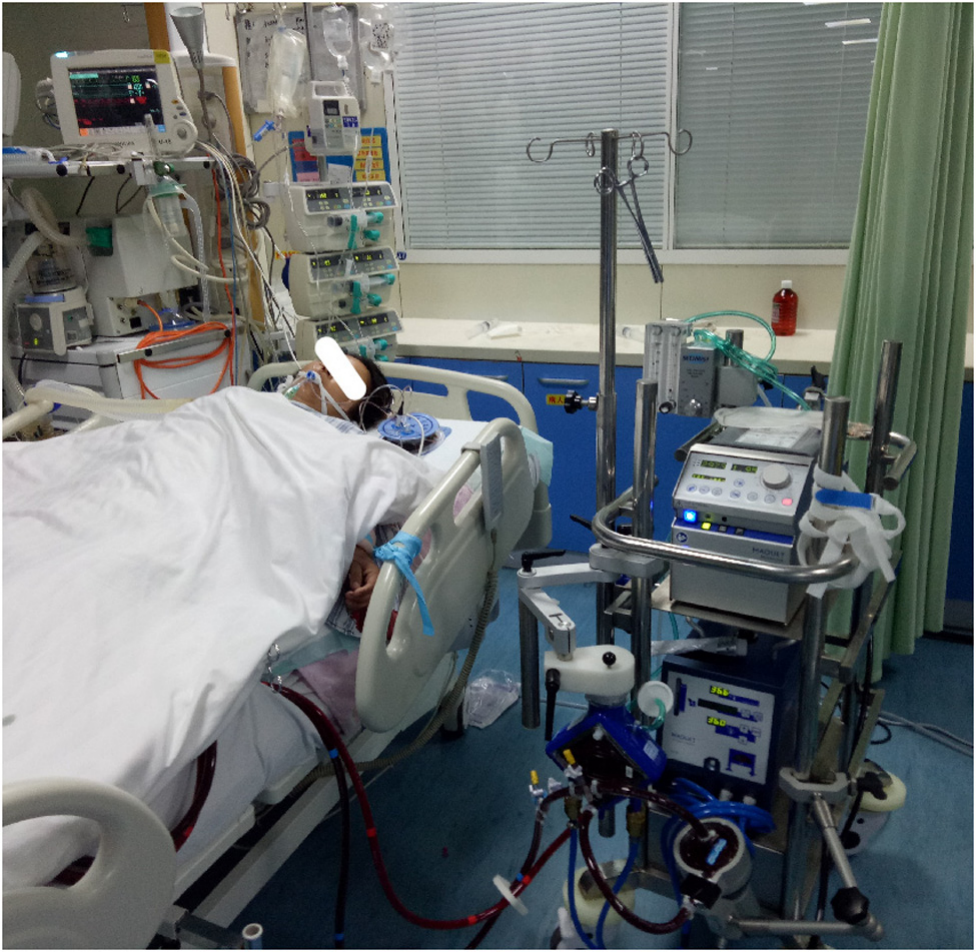
Patient with PCC treatment by VA ECMO.

**Figure 3:**
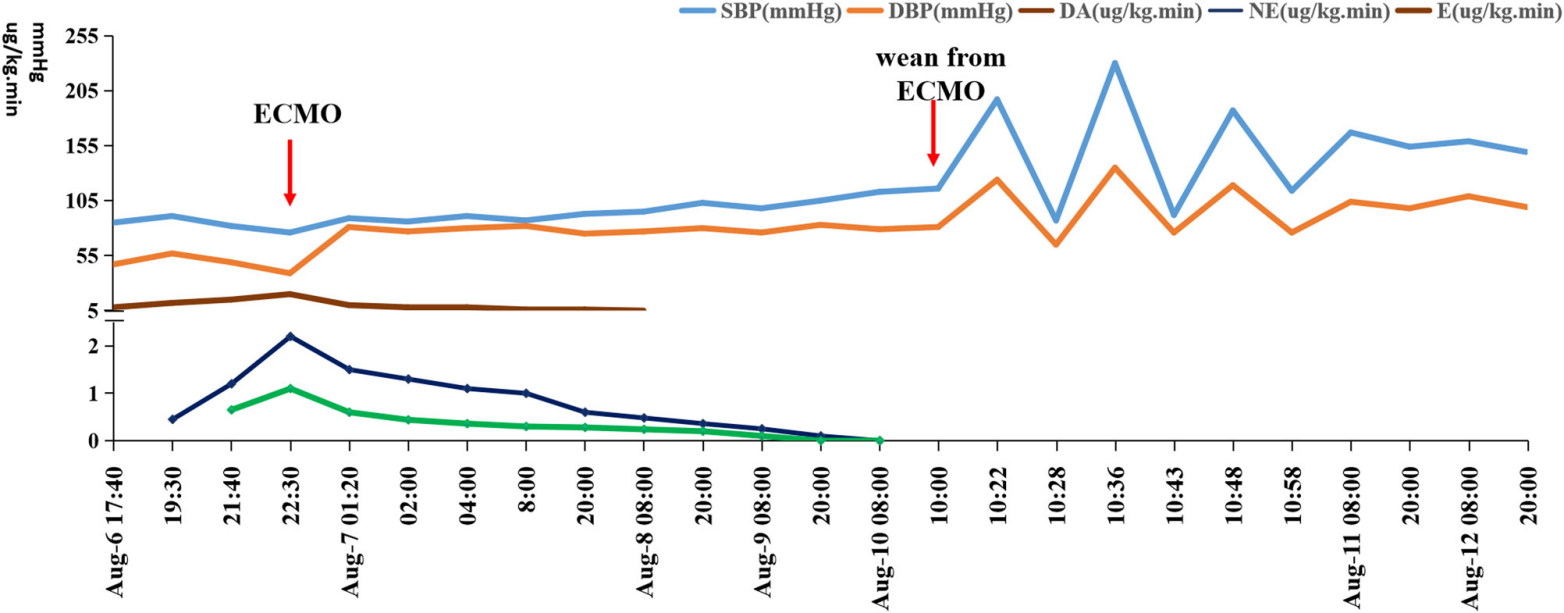
Dynamic changes in blood pressure and dosage of vasopressors in the patient. Systolic blood pressure (SBP), diastolic blood pressure (DBP), and dosages of norepinephrine (NE), epinephrine (E), dopamine (DA), and ECMO indicated. The dramatic cyclic blood pressure fluctuation during the catheter removed periods is particularly notable.

As cardiocirculatory system collapse was anticipated and the underlying diagnosis was considered treatable, we decided to perform VA ECMO for this patient; she then underwent bedside percutaneous right femoral arterial (17 F) and venous (21 F) cannulation using the Seldinger technique VA ECMO (Figure 2). The patient was connected to a crystalloid-primed ECMO circuit (Maquet Cardiopulmonary AG, Germany). The initial ECMO flow rate was 3.83 L/min to maintain the mean blood pressure at 80 mm Hg with less than 10 mm Hg of pulse pressure. Continuous venovenous haemodiafiltration was discontinued. The ECMO temperature was set at 35°C to decrease hyperthermia. Less than 6 h later, the patient’s vital signs were almost stable (T: 38.2°C, HR: 95 bpm, SPO_2_: 99%, BP: 86/78 mm Hg, norepinephrine: 0.43 µg/kg/min, and adrenaline: 0.24 µg/kg/min) with continuous ECMO and inotropic agent and vasopressor support; her urine output was approximately 200 cc/h. Over the next 3 days after ECMO was established, repeat TTE showed gradual improvement in biventricular function. Norepinephrine and adrenaline were withdrawn on the third day (Figure 3). The patient’s cardiac condition remarkably improved. TTE on day 4 with an ECMO flow rate of 2.0 L/min revealed normal to hyperdynamic biventricular activity (LVEF: 55%) without any regional wall motion abnormalities, and she was weaned from ECMO later on the same day.

**Figure 4:**
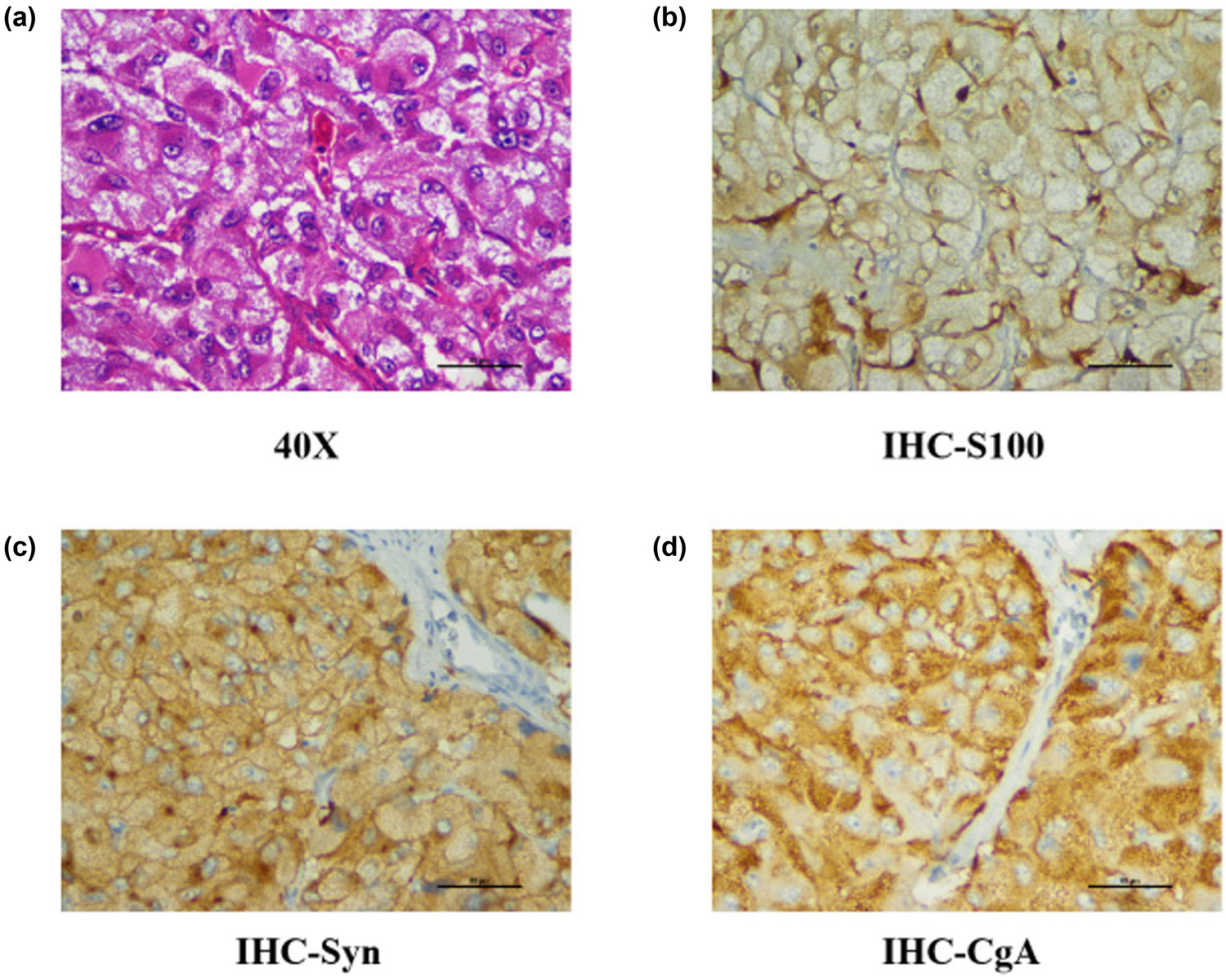
The histological diagnosis was pheochromocytoma of the adrenal medulla. (a) The tumor cells were distributed in nests under high magnification, with Sertoli cells and vascular sinuses visible around them. The tumor cells were polygonal and bubble-like with obvious nucleoli (HE ×400). (b) The expression of S-100 in the Sertoli cells (IHC ×400). (c) The expression of Syn in tumor cells (IHC ×400). (d) The expression of CgA in tumor cells (IHC ×400).


The patient’s blood pressure increased to 230/130 mm Hg when the catheter was removed; despite treatment with propofol and sufentanil at 200 mg/h and 50 µg separately, her blood pressure did improve and fluctuated from 130/60 mm Hg to 230/130 mm Hg in a short time. Intermittent sodium nitroprusside was required to control episodic hypertension; then, the diagnosis of phaeochromocytoma was suspected. Computed tomography revealed a left suprarenal tumour of 5.8 cm × 5.7 cm in size with central necrosis (Figure 1d). The vanillylmandelic acid concentration was 63.15 mg/24 h (normal value: 1.0–7.0 mg/24 h). A PCC diagnosis was made, and antihypertensive treatment with intravenous phentolamine and esmolol was gradually introduced in consultation with endocrinologists. The patient was transferred to the urology ward for preoperative preparation, and the tumour was eventually removed by laparoscopy. At last, pathology confirmed phaeochromocytoma (Figure 4). The patient was discharged from the hospital 1 week after the operation, and her blood pressure had returned to normal.

**Informed consent:** Informed consent has been obtained from all the individuals included in this study.
**Ethical approval:** The research related to human use has been complied with all the relevant national regulations, institutional policies, and in accordance with the tenets of the Helsinki Declaration, and has been approved by the Medical Ethics Committee of The First Affiliated Hospital of Wannan Medical College (Yijishan Hospital of Wannan Medical College).

## 3 Discussion

This phaeochromocytoma case demonstrates fulminant cardiomyopathy; however, the patient’s clinical manifestation gave no evidence of phaeochromocytoma diagnosis. Suspicion was first raised when the blood pressure fluctuated after the patient was weaned from ECMO and it was then confirmed by imaging and laboratory test. PCC has been defined as the release of large amounts of catecholamines resulting in severe organ dysfunction, especially haemodynamic instability and myocardial damage. PCC has been reported to mimic cardiomyopathy, myocardial infarction [3], pulmonary oedema, ARDS, sever haemoptysis [4], ischaemic or haemorrhagic stroke [5], acute kidney or liver injury, ischaemia of the ileus or bowel, metabolic disturbance, rhabdomyolysis, and thrombosis [6]. As far as we know, we are the first to report a patient with cardiogenic shock induced by PPC mimicking hyperthyroidism, which was successfully resuscitated by VA ECMO. Additionally, the patient recovered almost completely.

The rate of PCC misdiagnosis is very high. In a French multicentre cohort study [7] of PCC, among 34 PCC patients, 32 were under no suspicion of PCC before the complication leading to ICU admission; thus, the misdiagnosis rate was greater than 94%, and 8 of the 34 PCC patients were correctly diagnosed only during post-mortem. These eight patients died before the diagnosis was made [8]. In the current case, after the patient was admitted to the hospital, she rapidly developed fulminant cardiopulmonary failure without typical paroxysmal hypertension; although her thyroid hormone levels were not very high, and she did not show the characteristic clinical manifestations or thyroid gland sonographic findings of hyperthyroidism. High-quality research [9] has demonstrated that high thyroid hormone levels are not necessary to diagnose a thyroid storm, although with numerous doubtful points, which we chose to ignore. So, while “common things are common,” keeping an open mind regarding other possible differential diagnoses is essential in unusual cases [10]. Additionally, phaeochromocytoma should be considered in patients presenting with severe hemodynamic instability, particularly young patients [11].

An acute catecholamine crisis may be fatal before it is diagnosed [12]. Previous studies showed more than 85% mortality rate in the pheochromocytoma crisis [13]. The mortality rate of multisystem PCC was 45% based on a systematic review of 11 cases [14]. Numerous reports indicate that most patients with PCC crisis eventually die of heart failure and pulmonary oedema before diagnosis. And it does not respond to conventional treatment. Therefore, ECMO may be a lifesaving measure in these patients. Pheochromocytoma-related refractory cardiogenic shock requiring ECMO support has been reported by Haake et al. [15] and Suh et al. [16]. Previous reports have shown that ECMO, as an auxiliary means of cardiac resuscitation, can also achieve good results in patients with cardiac arrest [17]. Once ECMO is established, patients tend to improve quickly in a short time; it enables the use of alpha-blockers and fluid resuscitation, a contraindication because of the sustained hypotension and poor heart function. Therefore, this combination of hypotension and pathologic impairment treatment should achieve sustainable medical control in most cases. Mechanical circulatory support has a 92% survival rate of type B crisis. However, the control group had a 56% mortality rate, as described by Whitelaw et al. [18]. Since 2008, there have been frequent reports of the successful use of such circulatory support, as in the paper by Chao et al. [19] titled “Pheochromocytoma crisis is an absolute indication for ECMO.”

## 4 Conclusion

PCC mimics many conditions and is thus a masked killer. The misdiagnosis ratio was very high. For patients with unexplained cardiovascular impairment, especially in younger patients, we should consider the PCC.
